# Determination of Isosorbide-5-Mononitrate in Human Plasma by High-Performance Liquid Chromatography-Tandem Mass Spectrometry and Its Application to a Bioequivalence Study

**DOI:** 10.1155/2020/1753265

**Published:** 2020-07-17

**Authors:** Yinping Zhou, Aijing Liu, Ranran Jia, Mengyi Wu, Ni Wu, Chunyan Liu, Zhihui Han, Haitang Hu, Hongyun Wang, Qing He

**Affiliations:** ^1^Clinical Pharmacology Research Center, Peking Union Medical College Hospital, Chinese Academy of Medical Sciences and Peking Union Medical College, Beijing Key Laboratory of Clinical PK and PD Investigation for Innovative Drugs, Beijing 100730, China; ^2^Livzon Group Medical Clinical Research Center, Zhuhai 519020, China; ^3^Wuxi People's Hospital of Nanjing Medical University, Wuxi 214023, China

## Abstract

Isosorbide-5-mononitrate (5-ISMN), an organic nitrate vasodilator, has been widely used worldwide to prevent angina pectoris for more than two decades. A simple and sensitive high-performance liquid chromatography-tandem mass spectrometry (HPLC-MS/MS) method was developed and validated for the determination of 5-ISMN in human plasma. ^13^C_6_-5-ISMN is an internal standard, and 5-ISMN was extracted from human plasma (50 *µ*L) with ethyl acetate (200 *µ*L) by a simple liquid-liquid extraction method. The chromatographic separation was carried out on LC-20A (Shimadzu, Japan) using an analytical column ZORBAX XDB-C_18_ (4.6 × 50 mm, 5 *µ*m), coupled with API 4000 tandem mass spectrometers in a multiple reaction monitoring (MRM) mode. The mobile phase was composed of acetonitrile (organic phase A) and 2 mM ammonium acetate in water (aqueous phase B) with an isocratic elution of A/B = 90 : 10 (*v*/*v*). The total run time was 3.5 min with a small injection volume (5 *µ*L). This method was fully validated in every aspect of selectivity, linearity, accuracy, precision, matrix effect, extraction recovery, and different stabilities. It was proved that the calibration standards within the 5.00–1000 ng/mL concentration range were linear. The lower limit of quantification was 5.00 ng/mL for 5-ISMN. The intrabatch and interbatch accuracy (RE) ranged from −8.8% to 7.1% with precision between 2.4% and 6.6%. The mean values of 5-ISMN extraction recovery and matrix effect were 87.0% and 102.0%, respectively. The fully validated method was successfully applied for a bioequivalence clinical trial of oral 20 mg 5-ISMN tablets in healthy Chinese subjects.

## 1. Introduction

Isosorbide-5-mononitrate (5-ISMN) is an organic nitrate vasodilator and one of the long-acting metabolites of isosorbide dinitrate [1]. 5-ISMN is orally administered with an elimination half-life of 5 hours and is not subject to first-pass metabolism. 5-ISMN enters the systemic circulation together in the form of the original drug with high bioavailability [2]. 5-ISMN has been extensively used for cardiovascular diseases such as coronary heart disease and angina pectoris to prevent or at least reduce the occurrence of angina for many years [3], and it has been developed into various formulations such as tablets, immediate-release formulations, and sustained-release formulations [4]. Published analytical methods such as gas chromatography-mass spectrometry (GC-MS) [5–8] and liquid chromatography-tandem mass spectrometry (LC-MS/MS) [9–13] have been established for the identification or quantification of parent isosorbide dinitrate (ISDN) and its two active metabolites isosorbide-2-dinitrate (2-ISMN) or isosorbide-5-monoitrate (5-ISMN) in previous years. However, these assay methods presented some disadvantages such as time-consuming (long-term operation of more than 20 minutes [6] or tedious derivatization [7, 8]), low sensitivity (20 ng/mL [11] or 10 ng/mL [12]), requiring a large amount of plasma samples (0.2–0.5 mL) [11, 12] or reagents for sample preparation (3-4 mL) [9, 11], or requiring relatively expensive solid-phase extraction (SPE) accompanied with an LLOQ of 9.02 ng/mL [10], which may result in limited application to clinical studies. The mostly reported LC-MS methods to determine 5-ISMN showed relatively low sensitivity (the LLOQ of 10–20 ng/mL) [9–13] except for the report by Sun et al. [9]. Sun et al. [9] reported the LC-MS method with an LLOQ of 1.04 ng/mL, but it required a large amount of plasma (0.5 mL) and reagents (3 mL) for the sample preparation [9]. So, the fast and reliable 5-ISMN identification method is critical for clinical pharmacokinetic evaluation and clinical application.

In the article, we established a simple and robust HPLC-MS/MS method for the qualification of 5-ISMN in human plasma. This analytical method had two major features which were as follows: (1) only requiring 50 *μ*L of plasma sample with the LLOQ (5 ng/mL), especially suitable and convenient for clinical studies of patients, and (2) only a small volume (200 *µ*L) of ethyl acetate is used for sample pretreatment. Besides, all the reported LC-MS methods did not consider the precision and accuracy of hyperlipidemia/hemolysis and the stability of whole blood, which may be important in the study of 5-ISMN in patients or in BE studies containing fed condition. These reliable indicators were performed in our study. The analytical method was abundantly validated and proved to be specific and reproducible, and finally, it was successfully applied to determine the drug concentration of 5-ISMN in the bioequivalence clinical trial of oral 20 mg 5-ISMN tablets in healthy Chinese subjects.

## 2. Materials and Methods

### 2.1. Chemicals and Reagents

**Figure 1:**
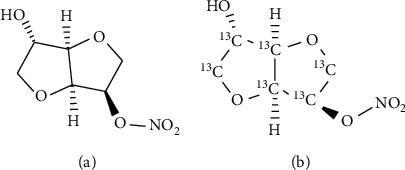
Structure of 5-ISMN (a) and ^13^C_6_-5-ISMN (b).

The standard of 5-ISMN was obtained from the National Institutes for Food and Drug Control (purity 99.9%, China), and the standard of ^13^C_6_-5-ISMN (called internal standard, IS) was gained from TLC Pharmaceutical Standards Ltd. (purity 99.0%, Canada) (Figure 1). Both acetonitrile and ethyl acetate with HPLC grade were bought from Honeywell Burdick and Jackson (Muskegon, ML, USA). Ammonium acetate (A.R. grade) was purchased from Beijing Chemical Reagents Company (China). However, deionized water was prepared using a Milli-Q purifying system (Millipore, Bedford, USA). Drug-free human plasma was obtained from six healthy donors in Peking Union Medical College Hospital.

### 2.2. HPLC Conditions

The chromatographic separation was carried out on LC-20A (Shimadzu, Japan) using an HPLC column ZORBAX XDB-C_18_ (4.6 × 50 mm, 5 *µ*m) and a guard column (ZORBAX Eclipse XDB-C_18_ 4.6 × 12.5 mm, 5 *µ*m). The mobile phase was composed of acetonitrile (organic phase A) and 2 mM ammonium acetate in water (aqueous phase B) with an isocratic elution of A/B = 90 : 10 (*v*/*v*). The autosampler syringe and the injection valve were washed sequentially by acetonitrile-deionized water at a ratio of 90 : 10 (*v*/*v*) to decrease the potential carryover. The autosampler temperature was set at 4°C. For each sample, the analytical run time was 3.5 min accompanied by a small injection volume (5 *µ*L), as well as the flow rate of 0.35 mL/min.

### 2.3. Mass Spectrometer Conditions

**Figure 2:**
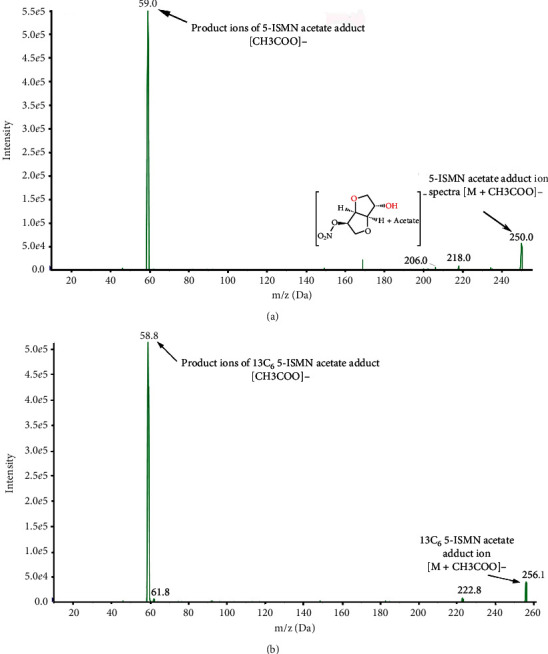
Product ion spectra of 5-ISMN (a) and IS (^13^C_6_-5-ISMN) (b).

The mass spectrometric detection was performed by an API 4000 mass spectrometer (AB SCIEX) under negative electrospray ionization (ESI^−^) source condition. The detection was operated in the multiple reaction monitoring (MRM) scan mode to analyze 5-ISMN and ^13^C_6_-5-ISMN (m/z 250.0 ⟶ 59.0 and m/z 256.1 ⟶ 58.8, respectively) (Figure 2). Furthermore, the optimized mass spectrometric parameters covered ion spray voltage of 4500 V, GS1 and GS2 at 50, declustering potential of 25, entrance potential of 10, collision energy of 20, and collision exit potential of 3, which are mainly presented in Table 1.

### 2.4. Calibration Standards and Quality Control Samples

Stock solutions of 5-ISMN (analyte) and its internal standard (^13^C_6_-5-ISMN) at the concentration of 0.5 mg/mL were prepared by acetonitrile-water (50 : 50, *v*/*v*). Stock solutions of 5-ISMN in duplicate were used for calibration standards and quality control samples (QCs) in human plasma matrix separately. First, the stock solutions were further diluted to the subsequent working solutions. The IS (^13^C_6_-5-ISMN) working solution was gained by mixing 20 *μ*L of the stock solution (0.5 mg/mL) with 9980 *μ*L of acetonitrile-2 mM ammonium acetate (50 : 50, *v*/*v*) to obtain a concentration of 1000 ng/mL. Then, for each analytical run, calibration standards were freshly prepared by sequentially adding the required amount of analyte working solutions to the drug-free human plasma, to achieve the concentrations of 5.00, 10.0, 25.0, 50.0, 100, 250, 500, and 1000 ng/mL. Low-, medium-, and high-quality control samples (called LQC, MQC, and HQC, respectively) were prepared in each batch at concentrations of 5.00, 8.00, 80.0, and 800 ng/mL, respectively. Finally, all the solutions and plasma samples above were aliquoted and frozen at −80°C or −30°C for different analytical conditions.

### 2.5. Preparation of Hyperlipidemia Matrix

In the European Bioanalysis Forum recommendations on the treatment of hyperlipidemia matrix [14], normal plasma spiked with triglycerides at a concentration of at least 300 mg/dl showed a milky appearance, which was defined as hyperlipidemia plasma matrices. Intralipid (20% emulsion) was used to prepare hyperlipidemia plasma matrices. So, this processed method was prepared as follows: 60 *µ*L intralipid and 3940 *µ*L of blank plasma were successively added to an Eppendorf tube and vortexed well. LQC, MQC, and HQC samples of 5-ISMN were prepared by the above matrix to assess hyperlipidemia precision and accuracy.

### 2.6. Preparation of 2% Hemolysis Matrix

According to the recommendation of the European Bioanalysis Forum [14], the hemolyzed plasma matrix was prepared by the addition of 2% hemolyzed blood to normal plasma (*v*/*v*). The process was described in detail as follows.

The 2% hemolysis matrix was prepared as follows: freshly blank whole blood collected from 6 sources was stored at −80°C for at least one hour, then ultrasonicated at room temperature for 30 min, and vortexed for 1 min to fully rupture blood cells to obtain hemolyzed whole blood. Finally, 200 *µ*L of hemolyzed whole blood was placed into 9800 *µ*L nonhemolytic blank plasma and mixed well to obtain a bright red 2% hemolysis matrix. QC samples at three concentration levels of 5-ISMN were obtained by the above 2% hemolysis matrix to investigate hemolysis precision and accuracy.

### 2.7. Sample Pretreatment

Before homogenization by vortex-mixing, frozen plasma samples were thawed thoroughly at room temperature. All the calibration standard samples, QC samples, and even clinical samples were extracted by the easy and feasible liquid-liquid extraction (LLE) method. The volume of 50 *µ*L plasma was placed into an Eppendorf tube including 200 *µ*L of ethyl acetate and 50 *µ*L of IS working solution (1000 ng/mL ^13^C_6_-5-ISMN). This mixture was vortex-mixed in 1 min and then centrifuged for 5 min at 13,000 rpm to extract the target analyte. The supernatant (100 *µ*L) was aspirated and evaporated to become dry with stable nitrogen stream for 10 minutes at room temperature. The residue was reconstituted with 200 *µ*L of mobile phase which was comprised of acetonitrile-2 mM ammonium acetate in water (90 : 10 *v*/*v*).

### 2.8. Method Validation

The developed method was completely validated in accordance with the US Food and Drug Administration (FDA) guidance for biological method validation [15] and European Medicines Agency Guideline on bioanalytical method validation [16].

#### 2.8.1. Selectivity

Selectivity referred to the chromatograms of at least six individual blank human plasma samples (double blank) to assess endogenous interferences of the plasma matrix based on the corresponding retention time of analyte and IS.

#### 2.8.2. LLOQ and Linearity

Lower limit of quantification (LLOQ) was described as the lowest concentration on the calibration curve and represented the sensitivity of the analytical method. Standard samples with eight different concentrations of 5-ISMN (5.00–1000 ng/mL) constituted a calibration curve, of which linear relationship was accessed by the correlation coefficient (*r*) and relative standard deviation (RSD) on at least three independent batches.

#### 2.8.3. Precision and Accuracy

Precision and accuracy on three consecutive batches completed in no less than two days were evaluated by 6 replicates at concentration level of LLOQ, LQC (low control sample), MQC (medium control sample), and HQC (high control sample) (5.00, 8.00, 80.0, and 800 ng/mL). At interbatch and intrabatch accuracy, no less than 75% of the back-calculated concentration must be in the range of 85–115% of its nominal concentration (80–120% at LLOQ).

#### 2.8.4. Extraction Recovery and Matrix Effect

The extraction recovery and matrix effect were evaluated by comparing the average peak areas of analyte and IS between LQC, MQC, and HQC (8.00, 80.0, and 800 ng/mL) quality control sample groups (plasma matrixes from 6 different individuals): group A (QC), extracted QC samples; group B (MQ), extracted blank matrix spiked with the corresponding concentrations of analyte and IS solutions; and group C (SQ), pure solution at equivalent concentrations as group A. Extraction recovery was calculated by QC/MQ. The absolute matrix effect was assessed by the matrix factor, which was calculated by MQ/SQ. Then, IS-normalized matrix effect was further calculated by the ratio of matrix factors between analyte and IS.

#### 2.8.5. Carryover

Carryover was at least evaluated by injecting a pretreated blank plasma sample after injecting the ULOQ sample of the first calibration curve in each analysis batch.

#### 2.8.6. Batch Capacity and Dilution Integrity

Batch capacity was confirmed by repeated injection of quality control samples to determine the maximum number of samples in an analysis batch. Taking into account some unknown sample concentrations over ULOQ, dilution integrity was investigated with a 10-fold dilution factor by using blank plasma to dilute 1600 ng/mL plasma sample of 5-ISMN to 160 ng/mL plasma sample (called dilution QC).

#### 2.8.7. Stability

The stability of 5-ISMN must be evaluated at each step prior to the determination of the clinical samples to simulate as much as possible the various storage and analysis conditions of clinical samples. These stability conditions mainly included whole blood stability, autosampler stability, and repeated injection reproducibility of pretreated plasma samples, as well as short-term and long-term stability and three freeze-thaw cycles of plasma samples in different temperature. Besides, stability of stock solution and working solutions at room temperature or under freezing conditions (−80^o^C) were also carried out.

#### 2.8.8. Accuracy and Precision of Hyperlipidemic/Hemolyzed Matrix

Considering the actual situation of clinical operation, freshly collected clinical samples may undergo hemolysis during storage or pretreatment, so it was necessary to assess the precision and accuracy of the analyte in the hemolysis matrix. Because of the bioequivalence evaluation of 5-ISMN containing fed trial, it was necessary to investigate the determination of the analyte influenced by the hyperlipidemia matrix.

### 2.9. Data Acquisition and Analysis

Data acquisition and data management were run through Analyst (version 1.5.1, AB SCIEX, USA) and Watson LIMS software (version 7.3, Thermo Scientific), respectively. The linear regression of calibration curves was weighted by 1/*x*^2^. The concentrations of unknown samples were determined by interpolation from two calibration curves in each batch. The noncompartmental method operated on WinNonlin (version 7.0, Pharsight, USA) was used to analyze relevant pharmacokinetic parameters of 5-ISMN.

### 2.10. Application for a Bioequivalence Study

The bioequivalence study of isosorbide 5-mononitrate (5-ISMN) tablets in healthy Chinese people was under fasting and fed conditions, respectively. This bioequivalence study was designed as an open, single-center, single-dose, two-cycle, two-sequence, randomized, crossover trial. The study protocol was approved by the Ethics Committee of People's Hospital of Wuxi (Jiangsu, China), and all the subjects signed informed consent before the study. A total of 56 healthy subjects participated in the study and received a single dose of 20 mg 5-ISMN tablets. Blood samples were obtained after administrating the 20 mg 5-ISMN tablet at specific time points within 36 h. Each subject had 19 blood collection points for the fasting condition and 18 blood collection points for the fed condition per cycle. To obtain plasma samples, the blood samples were drawn into tubes including anticoagulant and then centrifuged at 3000 rpm at 4°C within 10 min to take the supernatant. The above clinical plasma samples were frozen under −80°C condition before analysis.

### 2.11. Incurred Sample Reanalysis

According to the bioanalytical guidelines from the FDA [15] and the EMA [16], ISR is a necessary component of bioanalytical method validation as the calibration standards and QC samples in validation may not adequately represent the actual study samples. It is recommended that ISRs be conducted by repeating the analysis of several clinical samples in separate runs during the study. The ISR samples should be selected from the study samples around *C*_max_ and in the elimination phase. The difference of the results between the original study and the repeat study was determined with the following equation:

(1) $$\begin{array}{c} \text{Difference}\left(\text{\%}\right)=\frac{\left(\text{repeat}\mathrm{-ο}\text{riginal}\right)}{\text{mean}}\times 100\text{\%}. \end{array}$$

## 3. Results and Discussion

### 3.1. Development of the HPLC-MS/MS Method

In the article, a rapid, specific, and robust HPLC-MS/MS method was developed for the determination of 5-ISMN in human plasma. It was known that stable isotope-labeled compounds had similar structure and physicochemical properties to target analytes to reduce matrix effects; therefore, ^13^C_6_-5-ISMN was used for the internal standard to more accurately quantify the concentration of 5-ISMN. During the exploration of mass spectrometry conditions, an API 4000 mass spectrometer (AB SCIEX, USA) was used to detect the target analytes with the positive and negative ionization mode (ESI^+^ or ESI^−^). After the MS parameters reached the optimal conditions, it was turned out to be weak or inconsistent response at m/z 192.1 [M + H] or 190.1 [M−H], using 5-ISMN solution in acetonitrile or acetonitrile/water (*v*/*v*, 1 : 1). We consulted the relevant literature, such as the reports published by Silva [11] and Jain [13]. And we tried the possibility of quantification by 5-ISMN-adduct. It was found that the compound did easily form 5-ISMN adduct with different solutions, which may be related to the structure of the drug. In order to gain intense response, [M + X]^−^ adduct ion in the MS/MS transitions was gained by different solutions of 5-ISMN containing various anion additives (buffer), such as formic acid, acetic acid, ammonium acetate, and ammonia. Comparing responses of different additive ions, the 5-ISMN acetate adduct had the most stable and intense signal. Therefore, optimizing MS parameters in the meantime, the MS/MS transitions of 5-ISMN acetate adduct (250.0 ⟶ 59.0) and IS (^13^C_6_-5-ISMN adduct, 256.1 ⟶ 58.8) were selected to ensure high selectivity and efficiency in the MRM scan mode rather than in the SIM scan mode.

Chromatographic separation was constructed on LC-20A (Shimadzu, Japan) equipped with an API 4000 mass spectrometer. During method development, ZORBAX XDB-C_18_ was a commonly HPLC column for the widest range of compound species. After changing the different ratios of mobile phases and different flow rates, it turned out that 5-ISMN and IS had excellent peak shape and retention time when ZORBAX XDB-C_18_ (4.6 × 50 mm, 5 *μ*m) was accompanied by a mobile phase consisting of acetonitrile-2 mM ammonium acetate (90 : 10, *v*/*v*). In addition, a guard column was linked with the HPLC column to ensure a better peak shape.

### 3.2. Selectivity and LLOQ

**Figure 3:**
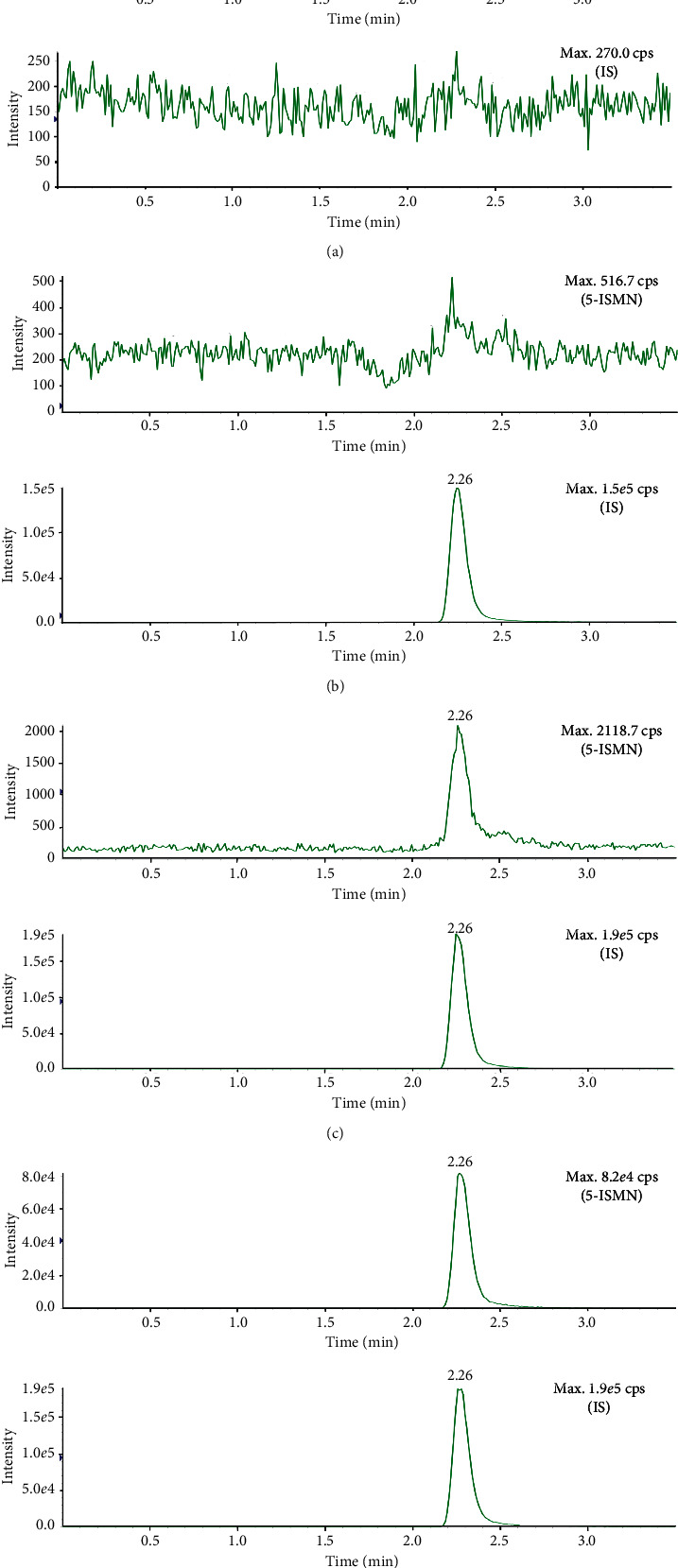
Representative MRM chromatogram of 5-ISMN and IS (^13^C_6_-5-ISMN, 1000 ng/mL). (a) Blank plasma, (b) blank plasma spiked with IS, (c) LLOQ plasma sample, and (d) unknown clinical plasma sample.

Figure 3 depicts the representative MRM chromatograms about blank samples without the analyte and IS, blank samples with IS only, LLOQ sample, and a clinical sample. Briefly, the interference of the blank sample was not beyond 14.7% of the average peak area of LLOQ at the corresponding retention time of the analyte, which was consistent with the acceptance criteria and indicated that the LLOQ (5.00 ng/mL) for 5-ISMN was suitable.

### 3.3. Linearity

Calibration curves were established within the concentration ranges of 5.00–1000.00 ng/mL for 5-ISMN in plasma samples. Correlation coefficients (r) over 0.99 with RSD 0.2% proved that all the calibration curves had an excellent linear relationship (Supplementary Figure 1 and Supplementary Table 1).

### 3.4. Precision and Accuracy

Intraday and interday precision and accuracy of the analytical method were determined by testing different concentrations of independent QC samples in plasma. As the specific results are shown in Table 2, the deviation from nominal concentration (precision or RSD) ranged from 2.4% to 6.6% and accuracy was between −8.8% and 7.1%, meeting the acceptance criteria.

### 3.5. Extraction Recovery and Matrix Effect

After a pretreatment method of liquid-liquid extraction using ethyl acetate, the mean extraction recovery of 5-ISMN at three QC levels was 87.0% with 7.0% RSD and that of IS (^13^C_6_-5-ISMN) was 80.9% with 9.7% RSD. The matrix effect was calculated by the peak area ratio of the analyte spiked with extracted blank matrix, to pure solution of equivalent concentrations. The matrix effect of 5-ISMN of LQC, MQC, and HQC samples ranged from 87.0% to 95.5% and the IS-normalized matrix effect of 5-ISMN was 98.6% to 107.8% under the present analytical condition.  Table 3 summarizes all the detailed results of extraction recovery and matrix effects, which demonstrated good extraction rates and no significant matrix effects indicating the rationality and superiority of liquid-liquid extraction method.

### 3.6. Carryover

For carryover evaluation, the peak area of 5-ISMN and ^13^C_6_-5-ISMN (IS) in the blank plasma sample following the ULOQ (upper limit of quantification) sample did not exceed 6.2% and 5.0% of the peak area of LLOQ, respectively, indicating no significant carryover.

### 3.7. Batch Capacity and Dilution Integrity

After repeated injections of LQC, MQC, and HQC samples of an eligible analytical batch for 9 times, the batch capacity of one analysis batch capable of analyzing the maximum number of samples was determined to be 162. This facilitated the efficient and rapid determination of the concentration of clinical samples.

Similarly, the result of the dilution integrity test presented that QC samples could be diluted with dilution factor 10 (mean values for precision did not exceed ±5.8% with an accuracy (RE) of −6.8%).

### 3.8. Stability

Table 4 presents the stability of the 5-ISMN plasma sample in kinds of situation. As a result, the average accuracy value of the LQC and HQC samples was within the range of -6.4%–7.8% of the normal values with the precision (RSD) not exceeding 7.1%. In briefly, plasma samples were kept stable at 25°C for 8 h, and whole blood samples before centrifugation were stable at 25°C for 2 h. Plasma samples were still stable at the autosampler (4°C) for 72 h after extraction and after three freeze-thaw cycles (from −80°C to RT). Repeated injection reproducibility of qualified batches after 64 hours in the autosampler was still feasible. Plasma samples for 91 days at −80°C and for 61 days at −30°C did not affect the assay results of 5-ISMN. In addition, the 5-ISMN and internal standard stock solutions (0.5 mg/mL) were stable at room temperature for 10 h and at −30°C for 63 days, and the 5-ISMN working solution remained stable under freezing condition (−80°C) for 63 days.

### 3.9. Precision and Accuracy of Hyperlipidemia Matrix and Hemolysis Matrix

As shown in Table 5, the accuracy and precision of 5-ISMN in the hyperlipidemia or hemolysis matrix were assessed by calibration curves prepared with normal blank samples spiked with 5-ISMN. The mean accuracy (RE) of LQC, MQC, and HQC samples in the 300 mg/dL hyperlipidemia matrix was −2.6%, 3.1%, and 3.8% with the precision (RSD) of 4.7%, 4.7%, and 2.8%, respectively. Besides, the mean accuracy of LQC, MQC, and HQC samples prepared with the 2% hemolysis matrix was −1.1%, 1.0%, and 6.5% with the precision (RSD) of 5.8%, 3.4%, and 1.7%, respectively. The above results indicated that the hyperlipidemia matrix and 2% hemolysis matrix did not affect the accurate determination of 5-ISMN of clinical uncertain samples. However, the accuracy and precision of hyperlipidemia or hemolysis matrices have never been studied in previous LC-MS/MS articles about 5-ISMN.

### 3.10. Application

**Figure 4:**
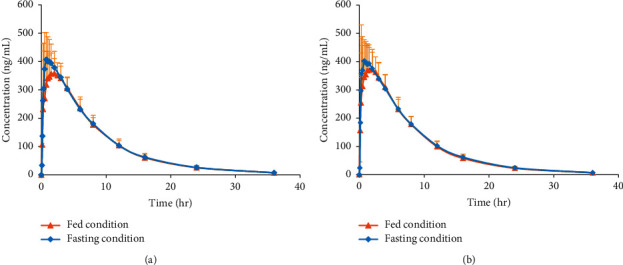
The mean concentration-time curves of reference and test drugs in Chinese healthy subjects after a single dose of the 20 mg 5-ISMN tablet in fasting condition (*n* = 28) (a) and fed condition (*n* = 28) (b).

The completely validated method was efficiently applied to the bioequivalence study of isosorbide 5-mononitrate (5-ISMN) tablets in healthy Chinese people under fasting and fed conditions, respectively. About 2,000 clinical samples from a total of 56 healthy subjects were efficiently analyzed in a week. Representative chromatogram of 5-ISMN in a clinical sample at 6 h after a single dose of 20 mg 5-ISMN is shown in Figure 3(d). The mean concentration-time curves of 5-ISMN of test and reference drugs in fasting and fed conditions are shown in Figure 4. It was found that food effect (a high-calorie diet) had no significant differences in the pharmacokinetic parameters of *C*_max_ and *T*_max_ for 5-ISMN, which was both consistent with the previous pharmacokinetic study [17] and the reported BE study about two 5-ISMN sustained-release drugs in 60 healthy Korean subjects on fasting and fed conditions [18].

### 3.11. Incurred Sample Reanalysis

**Figure 5:**
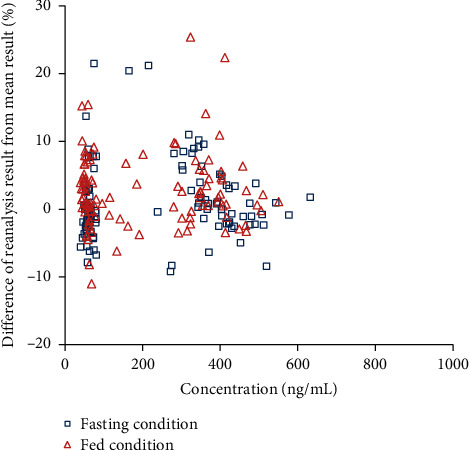
Percentage difference of reanalysis result versus mean result for 5-ISMN of clinical plasma samples in fasting condition (*n* = 105) and fed condition (*n* = 108).

105 clinical samples for 5-ISMN under the fasting condition and 108 clinical samples for 5-ISMN under the fed condition were reanalyzed, respectively. As shown in Figure 5, 97.1% of the samples for the fasting trial and 98.1% of the samples for the fed trial passed the criterion of percentage difference between reanalysis results and original results, indicating that the method was reliable and repeatable for determination of 5-ISMN in human plasma.

## 4. Conclusion

A sensitive and rapid LC-MS/MS method was developed and validated for the determination of 5-ISMN in human plasma. This method was practical and quickly applied to the bioequivalence study of oral 20 mg 5-ISMN tablets in Chinese volunteers.

## Figures and Tables

**Table 1:** The optimized mass spectrometric parameters.

Compound	Transition (m/z)	Declustering potential (DP)	Entrance potential (EP)	Collision energy (CE)	Collision cell exit potential (CXP)
5-ISMN	250.0 ⟶ 59.0	−25	−10	−20	−3
^13^C_6_-5-ISMN	256.1 ⟶ 58.8	−25	−10	−20	−3

**Table 2:** Intraday and interday accuracy and precision of quality control samples of 5-ISMN.

Nominal con. (ng/ml)	Intraday (*n* = 6)			Interday (*n* = 18)		
	Measured con. (mean ± SD, ng/mL)	Precision (RSD %)	Accuracy (%)	Measured con. (mean ± SD, ng/mL)	Precision (RSD %)	Accuracy (%)^a^
5	4.64 ± 0.23	4.8	-7.2	4.56 ± 0.30	6.6	−8.8
8	8.22 ± 0.44	5.3	2.8	8.13 ± 0.44	5.4	1.6
80	85.7 ± 4.01	4.7	7.1	82.2 ± 3.31	4.0	2.8
800	824 ± 31.2	3.8	3.0	817 ± 19.2	2.4	2.1

Concentration is in 3 significant figures; SD, standard deviation; RSD%, percent relative standard deviation (in one decimal place). ^a^Expressed as ((mean measured concentration−nominal concentration)/(nominal concentration)) × 100 (in one decimal place).

**Table 3:** Extraction recovery and matrix effect of 5-ISMN in plasma (*n* = 6).

Compound	Nominal con. (ng/mL)	Recovery (%) *n* = 6	Mean recovery% (RSD %)	Matrix effect (%) *n* = 6		IS-normalized matrix effect	Mean matrix effect% (RSD %)
				5-ISMN	IS		
5-ISMN	8	82.6	87.0 (7.0)	95.5	88.6	107.8	102.1 (0.85)
	80	84.3		87.0	88.3	98.6	
^13^C_6_-5-ISMN	800	93.8		93.4	94.3	99.8	
	1000	80.9	NA	NA	NA	NA	102.1 (0.67)

Concentration is in 3 significant figures; RSD%, percent relative standard deviation (in one decimal place). NA, not available.

**Table 4:** Stability assessments of 5-ISMN in plasma (*n* = 6).

Matrix	Storage conditions	Nominal con. (ng/mL)	Measured con. (mean ± SD, ng/mL)	Precision (RSD %)	Accuracy (%)^a^
Blood	Room temperature for 2 h	8	7.76 ± 0.18	2.3	−2.5
		200	212 ± 5.18	2.4	6.0

Plasma	Three circles of freeze/thaw	8	8.60 ± 0.35	4.1	7.5
		800	833.2 ± 8.82	1.1	4.1
	In autosampler for 72 h after extraction	8	8.62 ± 0.43	5.1	7.8
		800	823.2 ± 24.53	3.0	2.9
	Room temperature for 8 h	8	7.90 ± 0.35	4.5	−1.3
		800	823.5 ± 7.23	0.9	2.9
	Long-term stability (−30°C for 63 days)	8	7.49 ± 0.53	7.1	−6.4
		800	820.5 ± 26.85	3.3	2.6
	Long-term stability (−80°C for 91 days)	8	8.31 ± 0.19	2.2	4.0
		800	755.67 ± 9.93	1.3	−5.5
	Repeat injection for 64 h after extraction	8	7.71 ± 0.22	2.9	−3.6
		800	821.7 ± 25.12	3.1	2.7

Concentration is in 3 significant figures; SD, standard deviation; RSD%, percent relative standard deviation (in one decimal place). ^a^Expressed as ((mean measured concentration−nominal concentration)/(nominal concentration)) × 100 (in one decimal place).

**Table 5:** Precision and accuracy of 5-ISMN in hyperlipidemia and hemolysis matrices (*n* = 6).

Matrix	Nominal con. (ng/mL)	Measured con. (mean ± SD, ng/mL)	Precision (RSD %)	Accuracy (%)^a^
300 mg/dL hyperlipidemic matrix	8	7.79 ± 0.37	4.7	−2.6
	80	82.5 ± 3.89	4.7	3.1
	800	831 ± 23.18	2.8	3.8

2% hemolysis matrix	8	7.91 ± 0.46	5.8	−1.1
	80	80.8 ± 2.74	3.4	1.0
	800	852 ± 14.15	1.7	6.5

Concentration is in 3 significant figures; SD, standard deviation; RSD%, percent relative standard deviation. ^a^Expressed as ((mean measured concentration−nominal concentration)/(nominal concentration)) × 100 (in one decimal place).

## Data Availability

All data used to support the findings of this study will be made available from the corresponding author upon reasonable request.

## Supplementary Materials

Supplementary Materials Supplementary Figure 1 shows the representative calibration standards within the concentration ranges of 5.00–1000.00 ng/mL for 5-ISMN in plasma samples, and Supplementary Table 1 summarizes the correlation coefficients (*r*) over 0.99 with RSD 0.2%, so Supplementary Figure 1 and Supplementary Table 1 prove that all the calibration curves had an excellent linear relationship. Please see the specific data as follows.
