# Supplementary material for: Determination of Isosorbide-5-Mononitrate in Human Plasma by High-Performance Liquid Chromatography-Tandem Mass Spectrometry and Its Application to a Bioequivalence Study
Source: J Anal Methods Chem. 2020 Jul 17;2020:1753265. doi: 10.1155/2020/1753265 (PMC7382726; doi:10.1155/2020/1753265)
Supplement: Supplementary Materials — Supplementary Figure 1 shows the representative calibration standards within the concentration ranges of 5.00–1000.00 ng/mL for 5-ISMN in plasma samples, and Supplementary Table 1 summarizes the correlation coefficients (r) over 0.99 with RSD 0.2%, so Supplementary Figure 1 and Supplementary Table 1 prove that all the calibration curves had an excellent linear relationship. Please see the specific data as follows. [file 1753265.f1.docx]

Supplementary description：

Supplementary Figure 1 showed the representative calibration standards within the concentration ranges of 5.00-1000.00 ng/mL for 5-ISMN in plasma samples, and supplementary Table 1 summarized the correlation coefficients (r) over 0.99 with RSD 0.2%，so the *Supplementary Figure 1 and Supplementary Table 1* proved that all the calibration curves had an excellent linear relationship. Please see the specific data as follows.


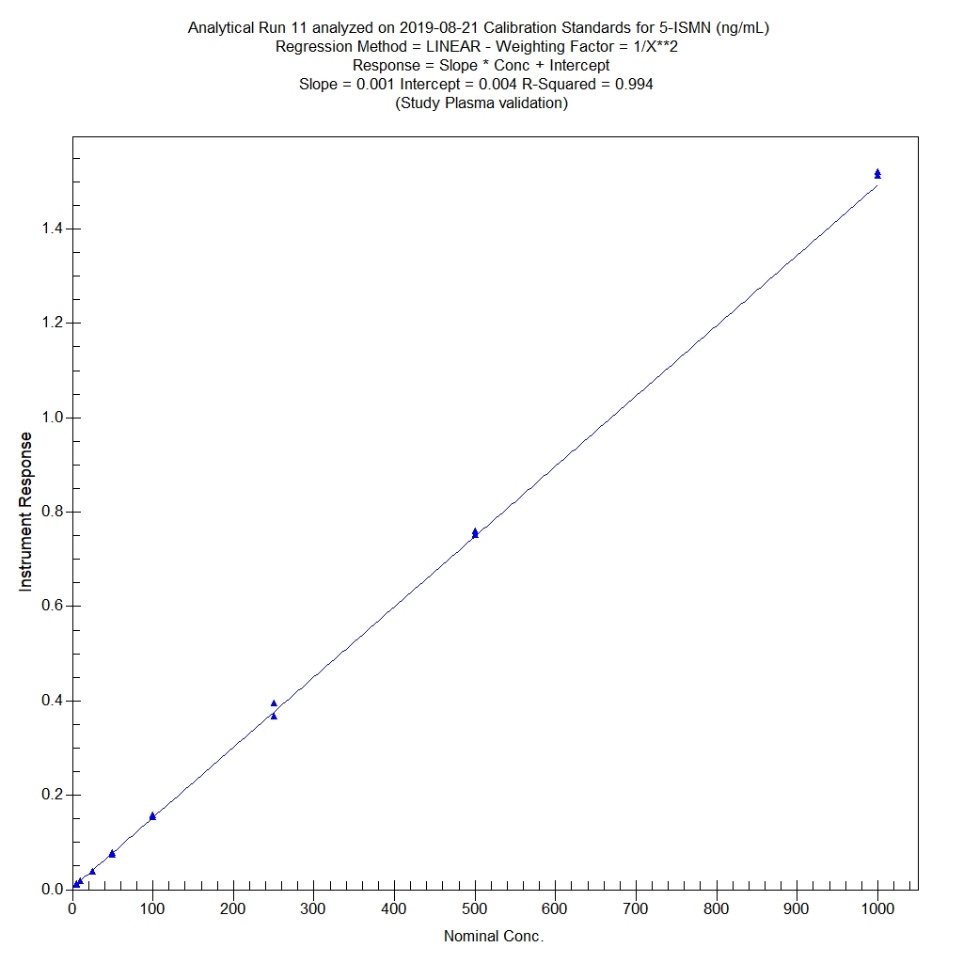


**Supplementary Figure 1:** the representative calibration standards of 5-ISMN

**Supplementary Table 1****:** Back-calculated concentrations of calibration standards

| Concentration | 5-ISMN (ng/mL) | | | | | | | | Slope | Intercept | r |
| --- | --- | --- | --- | --- | --- | --- | --- | --- | --- | --- | --- |
|  | 5 | 10 | 25 | 50 | 100 | 250 | 500 | 1000 |  |  |  |
| Mean | 5.03 | 9.99 | 24.4 | 48.7 | 103 | 254 | 510 | 976 | 0.002 | 0.002 | 0.997 |
| S.D. | 0.289 | 0.385 | 1.16 | 2.13 | 3.97 | 9.27 | 16.7 | 39.3 | 0.001 | 0.001 | 0.002 |
| %RSD | 5.7 | 3.9 | 4.8 | 4.4 | 3.9 | 3.6 | 3.3 | 4.0 | 50 | 50 | 0.2 |
| %RE | 0.6 | -0.1 | -2.4 | -2.6 | 3.0 | 1.6 | 2.0 | -2.4 |  |  |  |
| n | 28 | 27 | 28 | 28 | 27 | 27 | 28 | 28 | 14 | 14 | 14 |
